# Prevalence of neurological disorders in French bulldog: a retrospective study of 343 cases (2002–2016)

**DOI:** 10.1186/s12917-017-1132-2

**Published:** 2017-07-05

**Authors:** Vincent Mayousse, Loïc Desquilbet, Aurélien Jeandel, Stéphane Blot

**Affiliations:** 1 Université Paris-Est, Ecole Nationale Vétérinaire d’Alfort (EnvA), Unité de Neurologie, 7 avenue du général de Gaulle, 94700 Maisons-Alfort, France; 2 Université Paris-Est, Ecole Nationale Vétérinaire d’Alfort (EnvA), Unité de Biostatistiques et d’Epidémiologie Clinique, 7 avenue du général de Gaulle, 94700 Maisons-Alfort, France; 3 Inserm, IMRB U955-E10, 8 rue du général Sarrail, 94000 Créteil, France; 4 Present adress: Davies Veterinary Specialists, Manor Farm Buisiness Park, Higham Gobion, Herts SG5 3HR United Kingdom; 5 UMR BNMS Neurobiologie, Ecole Nationale Veterinaire d’Alfort, 7 avenue du General de Gaulle, 94700, Maisons Alfort, France

**Keywords:** Canine, Neurology, Epidemiology, Intervertebral disk disease, Referral centre, France

## Abstract

**Background:**

French Bulldog (FB) has significantly gained in popularity over the last few years, and seems to be frequently affected by various neurological conditions. The purpose of this retrospective study was to report the prevalences of neurological diseases in a large population of FB, presented with neurological signs between 2002 and 2016, and for which a definitive diagnosis was established. A secondary objective was to identify epidemiological characteristics regarding specific diseases in this singular breed.

**Results:**

During the study period, 533 FBs were presented for neurological signs, representing 18.7% of all admitted FBs (*N* = 2846). In total, 343 FBs with definitive diagnosis were included in this descriptive epidemiological study. Hansen type I intervertebral disk herniation (IVDH) was by far the most common neurological disorder (45.5% of all cases). The IVDH location was cervical in 39.8%, and thoracolumbar in 60.2% of cases. The median ages for cervical and thoracolumbar IVDH were 4.2 and 4 years, respectively. C3-C4 was the most commonly affected disk (57.8% of cervical IDVH) all locations combined. Spinal arachnoid diverticulum (SAD) was detected in 25 FBs, representing the second most common myelopathy (11.3%). A concurrent spinal abnormality was identified in 64.0% of SAD cases. Brain tumours represented 36.8% of encephalopathies, with glioma (confirmed or suspected) being the most common. Meningoencephalitis of unknown origin (MUO) represented 25.0% of brain disorders, females less than 5.5 years being more likely to be affected. Aside from central nervous system conditions, otitis interna associated with peripheral vestibular signs and bilateral congenital deafness (associated with white coat) were also common.

**Conclusions:**

The findings of this study suggest that FB seems to be prone to several neurological diseases. IVDH is clearly predominant in FB and cervical location seems more represented than in other breeds. FBs affected by IVDH tend to be younger than previously described, either for both cervical and thoracolumbar locations. Thoracic SAD was the second most common myelopathy, with a concurrent spinal anomaly identified in two thirds of the cases. MUO was more likely to affect young to middle-aged females. These findings could be of interest for owners, breeders, practicing veterinarians and insurance companies.

## Background

The French Bulldog (FB) is a canine breed originating from France. The numbers of FBs have markedly increased in recent decades. In Europe, FB has significantly gained in popularity over the last few years. For example, annual registrations of FBs have quadrupled over the past 15 years in France [1], and in the United Kingdom, 14,607 new registrations to the Kennel Club were recorded in 2015 versus 526 in 2006 [2]. In North America, FB was the 6th most popular breed in the United States in 2014 [3], and the 9th most popular breed in Canada in 2015 [4]. Due to the brachycephalic and chondrodystrophic body conformation resulting from selective inbreeding, a high prevalence of various diseases has been described in this breed, including several neurological conditions [5]. These include not only myelopathies such as compressive vertebral malformations [6], spinal arachnoid diverticula [7] and intervertebral disc disease [8], but also encephalopathies such as brain tumours [9] or non-infectious encephalitides [10]. To the authors’ knowledge, no study has yet reported the prevalences and distributions of different neurological disorders in FB or has described a specific neurological condition in this emerging breed. The primary objective of this epidemiological study was therefore to report the prevalences of different neurological conditions in a large population of FBs presented for neurological signs at a major referral centre. A secondary objective was to identify epidemiological characteristics regarding specific diseases in this singular breed.

## Methods

### Case selection

Case records of all French bulldogs presented for neurological signs (including spinal pain) at our institution between January 1st, 2002 and January 1st, 2016 were retrospectively reviewed. Dogs were included if they met all the following inclusion criteria: (i) neurological clinical signs, including isolated spinal pain, (ii) complete available records and (iii) a definitive etiological diagnosis. The only exception was the inclusion of young dogs presented for an auditory function screening, as several animals were asymptomatic. Dogs that did not present actual neurological clinical signs or signs mimicking a neurological condition but related to another cause (e.g. orthopaedic or ophthalmic conditions) were otherwise excluded. Similarly, animals with only a neuroanatomical diagnosis (e.g. “T2-L2 myelopathy”, or “*cauda equina* syndrome”) were not included in the study.

### Neurological diseases classification

When a dog was presented at our institution two times or more for neurological conditions, only the first one for which the dog was presented was taken into account. Similarly, when two concomitant neurological diseases were diagnosed at the same time on the same dog (i.e. with one being an incidental finding), only the one responsible for the clinical signs was retained. To facilitate data processing, each case was assigned to one of the following neuroanatomical categories, according to the definitive diagnosis: encephalopathy, myelopathy, peripheral nervous system (PNS) & muscles disorder, and unclassified neurological condition. Diseases unrelated to one of the above-listed categories, such as paroxysmal dyskinesia, tremors syndromes and congenital deafness were grouped together under “unclassified neurological conditions”.

### Criteria used for the diagnosis of specific diseases

Definitive diagnoses for each patient were then established by a board-certified neurologist based on patient’s signalment and history, clinical findings and appropriate ancillary tests, according to the current knowledge for each condition. Ancillary tests comprised miscellaneous blood testing (including biochemical analyses, serum bile acids measurement, complete blood count, electrolytes, hormonal testing, serology), cerebrospinal fluid (CSF) analysis, PCR screening for various endemic infectious agents of the nervous system, cytology and histology of various tissues, otoscopy, bacterial culture on various materials, muscles and nerves biopsies, myelography, cross-sectional imaging (Computed tomography [CT] and magnetic resonance imaging [MRI] scans), and electrodiagnostics (electromyography, nerve conduction studies and brainstem auditory evoked response [BAER]). Two MRI devices were used during the study period: a low-field (0.2 T) device prior to 2013, and a high-field device (1.5 T) after 2013. Criteria allowing diagnoses of diseases the most frequently expected are listed in the following sections.

#### Brain diseases

When available, brain tumours were diagnosed based on histological examination. In cases where a histological examination was not performed, MRI criteria were used. An intracranial glioma was defined as an intra-axial solitary lesion, more or less enhancing after paramagnetic intravenous contrast media administration, accompanied or not by surrounding oedema and/or mass effect [11]. Similarly, pituitary neoplasia diagnosis was based upon histological analysis and/or CT or MRI imaging features (masses well-delineated in the pituitary area, more or less invading the surrounding parenchyma with contrast enhancement), along with consistent biochemical or ultrasonographic abnormalities (hypercortisolism, bilateral adrenal enlargement etc. [12]). When the diagnosis was achieved through imaging criteria, the term “suspected neoplasia” was therefore used to refer to these diseases.

In cases of absent histopathological diagnosis, meningoencephalitis of unknown (MUO) origin was diagnosed based on the following previously described criteria: Focal or multifocal clinical signs of brain disease, T2-weighted multifocal intra-axial hyperintense lesions with variable T1-weighted contrast enhancement on MRI, mononucleated pleocytosis on CSF analysis, and exclusion of endemic infectious diseases [13]. Idiopathic epilepsy diagnosis was based upon the International Veterinary Epilepsy Task Force consensus, and included normal inter-ictal examination, normal brain MRI and CSF analysis, as well as a normal comprehensive biochemistry profile investigating metabolic causes of seizures, including serum bile acid and electrolytes [14]. Criteria allowing diagnosis of otogenic bacterial encephalitis included consistent central nervous system (CNS) clinical sings, middle/inner ear MRI and/or CT abnormalities, T1-weighted meningeal and/or brain parenchyma enhancement on MRI, inflammatory CSF (with or without bacterial culture), and a positive response to antibiotic treatment. The diagnosis of metabolic encephalopathies was based on clinical signs suggestive of brain disease, consistent MRI findings and an identified metabolic origin on blood analysis (e.g. hepatic or renal failure) [15].

#### Myelopathies

Intervertebral disk herniation was diagnosed either by CT, MRI or myelography associated with consistent clinical signs and onset. The distinction between Hansen type I and type II IVDH was based upon a combination of clinical and imaging criteria and the perioperative appearance of herniated material in dogs that underwent surgical treatment, as described in previous studies. Hansen type I IVDH was suspected on CT if hyperdense presumed disk material was observed in the intervertebral space and/or within the vertebral canal, along with subsequent spinal cord compression on transverse planes and/or epidural fat displacement [16]. It was suspected on MRI if the disk had extruded through the annulus fibrosus, and appeared as a compressive extradural hypointense (either in T2 or T1-weighted) single lesion, mostly lateralized and dispersed from either side of the intervertebral space [17]. Hansen type I was suspected during surgery if calcified/mineralized nucleus pulposus was extruded in the vertebral canal and/or under the dorsal longitudinal ligament [18].

Spinal arachnoid diverticulum (SAD) was defined on myelography or CT-myelography as contrast-filled, tear-drop shaped expansion of the subarachnoid space, with a possible abrupt interruption of the contrast column immediately after the lesion. On MRI, it was defined as a T2-weighted hyperintense, T1-weighted and/or FLAIR hypointense lesion of the subarachnoid space [19].

When histological examination was not performed, usual previously described imaging criteria were used to diagnose spinal tumours, especially regarding the relationship between the lesion and the subarachnoid space [20].

#### PNS & muscle disorders

Otitis interna was defined as the combination of clinical signs suggestive of a peripheral vestibular syndrome, evocative imaging findings either with CT (fluid-filled tympanic bulla) or MRI (fluid-filled tympanic bulla and loss of the normal T2-weighted hypersignal of the inner ear), and evidence of inflammation/infection on bulla cytology. Congenital deafness was defined as hearing loss or deficits since birth, confirmed by a consistent BAER study [21].

### Data acquisition and statistical analysis

The clinical database of the institution was searched using the clinical software (CLOVIS, 4Dv13) and appropriate keywords. When information was missing from numerical records, the paper files were retrieved if available. For each case fulfilling the inclusion criteria, data were recorded using a form created with EpiData v3.1 Software (Lauritsen J.M., Bruus M. & Myatt M., UK/Denmark). Information regarding file number, age, sex, body weight, duration of clinical signs, and definitive diagnosis were collected. Complete data were then exported into an Excel 2010 spreadsheet (Microsoft Office 2010, Excel 2010) for further statistical evaluation. Percentages for each subpopulation were calculated with a 95% confidence interval, using the asymptotic/Wald method (for groups in which n x *p* > 5, where n is the number of individuals in the concerned subpopulation, and p the estimate prevalence rate), or the exact binomial/Clopper-Pearson method (for groups in which n x *p* < 5, EpiTools, AusVet Animal Health Services). The χ^2^ statistical test (or Fisher’s exact test when appropriate) was used to assess the statistical association between age (taken as a binary variable with appropriate cut-off) or sex, and the occurrence of IVDH or meningoencephalitis of unknown origin. Odds Ratio (OR) were calculated to quantify the association between sex and occurrence of MUO, age and occurrence of IVDH, and were provided with their 95% confidence interval (CI). A *P-value* < 0.05 was considered significant. Statistical tests were performed using a website dedicated to statistical analysis (BiostaTGV, http://marne.u707.jussieu.fr/biostatgv/). A receiver operating characteristic (ROC) curve analysis was performed to determine a cut-off value for the age that best discriminated the presence (versus absence) of meningoencephalitis of unknown origin. The optimum cut-off value was determined with Youden’s index criteria. An appropriate statistical software was used for the ROC analysis (SAS Software, version 9.3).

## Results

### Study population

Between 2002 and 2016, 2846 FBs were presented at our institution (representing 3.1% of all dog breeds), all chief complaints and all departments combined. Over this period of time, FB was the sixth most popular breed after standard poodle (4.1% of all dogs breeds), German shepherd (4.2%), Labrador retriever (6.2%), Yorkshire terrier (6.8%) and mixed-breed dogs (10.1%). A total of 533 FBs (18.7% of the 2846 FBs) were presented for suspected neurological signs. During the same period, a total of 88,863 dogs (FB excluded) were presented at our institution, of which 10,150 (11.4%) were admitted for neurological clinical signs.

**Fig. 1:**
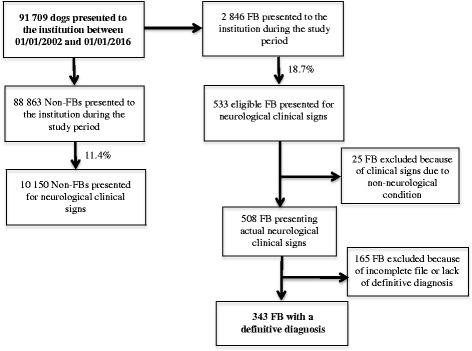
Data flow diagram illustrating the case selection procedure of the 343 French bulldogs (FB) selected from the general hospital population between 2002 and 2016, and presenting neurological clinical signs and a definitive diagnosis

Among the 533 FBs presented for neurological clinical signs, 25 were excluded because the clinical examination failed to identify a neurological disorder. A further 165 animals were excluded because of incomplete files and/or absence of definitive diagnosis. In the end, 343 FBs with a confirmed neurological disease and a precise diagnosis were included in the statistical analysis, representing 12% of all FBs presented to the institution during the study period. The whole case selection procedure is detailed in Fig. 1. Two concomitant neurological conditions were diagnosed in only 9 dogs at the time of presentation.

### Overall distribution of neurological diseases

In total, 64.7% of the 343 dogs presented a myelopathy (*n* = 222 dogs, 95% CI 59.7–69.8%), 19.8% presented an encephalopathy (*n* = 68, 95% CI 15.6–24.0%), 9.3% presented an unclassified condition (*n* = 32, 95% CI 6.5–12.9%) and 6.1% presented a PNS/muscle disorder (*n* = 21, 95% CI 3.8–9.2%). The median age of the FBs in this study was 4.0 years (range from 0.2 to 14.5 years). There were 188 males (including 19 castrated dogs) and 155 females (including 64 spayed females). All the results are detailed in Table 1.

**Table 1:** Distibution of the different neurological diseases in the 343 FBs from the study, with a definitive diagnosis

Category/Diseases	*N* = 343 (%)
**Myelopathies**	**222/343 (64.7%)**
IVDH	156/222 (70.3%), *representing 161 IV sites*
Cervical IVDH	64/161 (39.8%)
C3-C4	37/64 (57.8%)
C4-C5	12/64 (18.8%)
C2-C3	11/64 (17.2%)
C5-C6	4/64 (6.2%)
Non-cervical IVDH	97/161 (60.2%)
T13-L1	20/97 (20.6%)
L2-L3	17/97 (17.5%)
L3-L4	16/97 (16.5%)
L1-L2	14/97 (14.5%)
T12-T13	10/97 (10.3%)
L4-L5	10/97 (10.3%)
*Other locations*	10/97 (10.3%)
Spinal arachnoid diverticulum	25/222 (11.3%)
Thoracic	22/25 (88%)
Cervical	3/25 (12%)
Compressive vertebral malformations	19/222 (8.6%)
Hemivertebra + kyphosis	17/19 (89.5%)
*Other CVM*	2/19 (10.5%)
Spinal neoplasia	7/222 (3.1%)
Syringohydromyelia	6/222 (2.7%)
*Other myelopathies*	9/222 (4%)
**Encephalopathies**	**68/343 (19.8%)**
Brain neoplasia	25/68 (36.8%)
Glioma	17/25 (68%)
Pituitary neoplasia	5/25 (20%)
*Other neoplasias*	3/25 (12%)
MUO/optic neuritis	17/68 (25%)
Idiopathic epilepsy	9/68 (13.2%)
Infectious encephalitis	8/68 (11.8%)
Metabolic	3/68 (4.4%)
*Other encephalopathies*	6/68 (8.8%)
**Unclassified neurological conditions**	**32/343 (9.3%)**
Congenital deafness	29/32 (90.6%)
Bilateral	21/29 (72.4%)
Unilateral	8/29 (27.6%)
*Other unclassified conditions*	3/32 (9.4%)
**PNS/muscle disorders**	**21/343 (6.1%)**
Otitis interna/PVS	14/21 (66.7%)
Myopathies	3/21 (14.3%)
Idiopathic vestibular syndrome	2/21 (9.5%)
*Others PNS/muscle disorders*	2/21 (9.5%)

*FB* French bulldog, *IVDH* intervertebral disk herniation, *IV* intervertebral, *CVM* compressive vertebral malformation, *MUO* meningoencephalitis of unknown origin, *PNS* peripheral nervous system, *PVS* peripheral vestibular syndrome

### Myelopathies distribution

Hansen type I intervertebral disk herniation was the most common myelopathy, with 70.3% documented cases (*n* = 156 dogs, 95% CI 64.3–76.3%). IVDH accounted for 45.5% (95% CI 40.2–50.8%) of all the neurological conditions, and for 5.5% of all FBs presented to our institution during the study period (95% CI 4.6–6.3%). Five dogs presented two IVDH at the time of presentation, representing a total of 161 sites of disk herniation. Cervical IVDH accounted for 39.8% of all IVDH (*n* = 64, 95% CI 32.2–47.3%), whereas thoracolumbar and lumbar locations represented 60.2% of IVDH (*n* = 97, 95% CI 52.7–67.8%). There was no significant association between sex and IVDH (*p* = 0.95). When cervical and thoracic/lumbar IVDH were taken together, most dogs (80.8%, 95% CI 74.6–87.0%) were more than 3 years old. Age was significantly associated with IVDH, since 81% of FBs affected by IVDH were 3 years old or more, compared to 64% of dogs affected by another myelopathy (OR = 2.4, 95% CI 1.2–4.5, *p* < 0.01).

Spinal arachnoid diverticulum was the second most common myelopathy, with 11.3% of dogs affected by this condition (*n* = 25, 95% CI 7.4–16.2%). SAD represented 7.3% of all diseases of the study (95% CI 4.5–10.0%). Compressive vertebral malformation was diagnosed in 8.6% of dogs with myelopathy (*n* = 19, 95% CI 5.2–13.0%), followed by a neoplastic condition (either spinal or vertebral neoplasia) in 3.2% of dogs (*n* = 7, 95% CI 1.3–6.4%), syringomyelia in 2.7% of dogs (*n* = 6, 95% CI 1.0–5.8%), and ischemic myelopathy in 1.8% of dogs (*n* = 4, 95% CI 0.05–4.5%). The remaining disorders of the spinal cord were acute non-compressive nucleus pulposus extrusion (*n* = 3), immune-mediated myelitis (*n* = 1), and lumbosacral spinal cord dermoid sinus (*n* = 1).

#### Cervical intervertebral disk herniation

The median age of dogs affected by cervical IVDH was 4.2 years (range from 1.5 to 10 years), and the median weight was 12.4 kg (range from 8 to 19 kg). Cervical hyperesthesia, often pronounced, was observed in 82.8% of dogs presenting cervical IVDH (*n* = 53, 95% CI 73.5–92.0%). The most commonly affected site in the cervical region was C3-C4 (57.8% of cervical IVDH, *n* = 37, 95% CI 45.7–69.9%) followed by C4-C5 (18.8%, *n* = 12, 95% CI 10.1–30.5%) and C2-C3 (17.2%, *n* = 11, 95% CI 8.9–28.7%). An epidural hematoma/haemorrhage was observed in 3 cases that underwent cervical ventral slot surgery.

#### Thoracolumbar intervertebral disk herniation

Among these non-cervical IVDH (*n* = 97), 68 were located in the thoracolumbar (T3-L3) region and 29 were located in the lumbosacral (L4-S) region. The median age of FBs affected by thoracic and lumbar IVDH was 4 years (range from 1.7 to 13 years). The median weight was 12.4 (range from 4.5 to 18 kg). The most commonly affected intervertebral spaces in this group of dog were T13-L1 (20.6%, *n* = 20 95% CI 12.6–28.7%), followed by L2-L3 (17.5%, *n* = 17, 95% CI 10.0–25.1%), L3-L4 (16.5%, *n* = 16, 95% CI 9.1–23.9%) and L1-L2 (14.5%, *n* = 14, 95% CI 7.4–21.4%). Finally, T12-T13, L4-L5 and other intervertebral locations regrouped 10 dogs each (representing 10.3% of thoracolumbar IVDH each, 95% CI 4.3–16.4%). An extradural haemorrhage and/or hematoma was observed during cross-sectional imaging and/or surgery in 27.8% (*n* = 27, 95% CI 18.9–36.8%) of all cases diagnosed with thoracic or lumbar IVDH, regardless of the affected disk location.

#### Spinal arachnoid diverticulum

Concerning SAD, 88.0% (*n* = 22, 95% CI 75.3–100%) were located in the thoracolumbar (T3-L3) region. More than three quarters of the thoracolumbar SAD cases were located between T9 and T12 (77.3%, *n* = 17, 95% CI 59.8–94.8%). In the remaining 12.0% (*n* = 3, 95% CI 0.0–24.7%) of animals affected by this condition, a cervical (C1-C5) location of the SAD was observed. No SAD was found in the caudal cervical (C6-T2) or lumbosacral (L4-S) segments. In addition, a vertebral malformation (*n* = 12) or a mild IVDH (*n* = 4), at the level or distant from maximum 2 to 3 vertebral bodies to the SAD, was identified in 64.0% (*n* = 16, 95% CI 45.2–82.8%) of all cases. The median age of FBs affected by SAD was 4.5 years (range from 1 to 10.7 years).

#### Compressive vertebral malformations

Concerning the 19 dogs clinically affected by congenital vertebral malformations, 17 presented with hemivertebrae (89.5%, 95% CI 75.7–100%) associated with a major kyphosis responsible for a compression of the spinal cord. The most commonly affected vertebrae were T6, T7, T8, and T10 (with 3 abnormal vertebrae for each). Five FB presented with two or more malformations. One dog presented with a wedge-shaped vertebrae and one dog presented with L6-L7 *spina bifida*.

### Encephalopathies distribution

#### Brain neoplasia

A suspected brain neoplasia was observed in 36.8% of FBs affected by an encephalopathy (*n* = 25, 95% CI 28.6–53.3%), with a glioma, either suspected or confirmed, being the most common (*n* = 17, 68% of all neoplasias 95% CI 49.7–86.3%). Five FBs presented a pituitary macroadenoma (*n* = 5, 20.0% of neoplasias 95% CI 6.8–40.7%). The three remaining animals were affected by intracranial lymphoma, malignant intracranial peripheral nerve sheath tumour of cranial nerve III, or suspected osteosarcoma of the calvarium. Three gliomas and 2 pituitary macroadenomas were confirmed histologically (either with biopsies or post mortem examinations). The median age of FBs with brain neoplasia was 9.0 years old (range from 5 to 14.5 years).

#### Meningoencephalitis of unknown origin

Meningoencephalitis of unknown origin represented 25.0% of the encephalopathies (*n* = 17, 95% CI 15.3–37.0%). The median age of FBs affected by MUO was 2.25 years (range from 0.8 to 6.5 years). Among the 68 (19.8%) FBs that presented an encephalopathy, MUO was more frequently observed in females than in males (OR = 7.1, 95% CI 2.0–25.2, *p* < 0.01). ROC curve analysis enabled us to determine that the cut-off age that best discriminated the presence versus the absence of MUO was 5.5 years, with a sensitivity of 60.7% and a specificity of 94.1% (Area under the curve = 0.76). Therefore, by using this cut-off, 94.1% of FBs less than 5.5 years old presented with clinical signs related to an encephalopathy were affected by MUO, whereas 60.7% of FBs of 5.5 years old or more presented with the same clinical signs were not affected by this condition.

#### Other encephalopathies

Regarding other conditions, idiopathic epilepsy represented 13.2% of encephalopathies (*n* = 9, 95% CI 6.2–23.6%), and bacterial encephalitis associated with otitis media/interna 11.8% (*n* = 8, 95% CI 5.2–21.9%). The remaining encephalopathies were metabolic encephalopathies (*n* = 3, two hepatic encephalopathies, and one uraemic encephalopathy), congenital hydrocephalus (*n* = 2), cannabinoid intoxications (*n* = 2), ischemic stroke and degenerative encephalopathy (*n* = 1 for each condition).

### Other neurological conditions distribution

Among the unclassified neurological disorders (*n* = 32), 90.6% of FBs presented congenital deafness (*n* = 29, 95% CI 80.5–100.0%). Bilateral deafness was detected in 72.4% (*n* = 21, 95% CI 56.1–88.7%) of animals presenting congenital deafness, versus 27.6% for the unilateral form of the condition. Among all FBs presented with congenital deafness, 79.3% were white or had white in their coat (*n* = 23, 95% CI 64.6–94.1%). Idiopathic head tremors were observed in 2 animals, and narcolepsy/cataplexy in a single case.

Among PNS/muscles disorders (*n* = 21), 66.7% of animals presented an otitis interna with neurological signs (see below, *n* = 14, 95% CI 46.5–86.8%), 14.3% presented a myopathy (*n* = 3, 95% CI 3.0–36.3%), with one case of each the following conditions: immune-mediated polymyositis, ischaemic neuromyopathy and corticosteroids-induced myopathy. Two animals presented an idiopathic acute vestibular syndrome, one case presented a chronic steroid-responsive polyneuropathy, and one case presented a malignant peripheral nerve sheath tumour. Among the FBs affected by otitis media/interna, all animals were presented with a peripheral vestibular syndrome, eight cases had concurrent ipsilateral facial paralysis, and four cases had a concurrent Horner syndrome.

## Discussion

This study revealed that 18.7% of FBs admitted in our institution during the study period presented with neurological clinical signs, 12% when only dogs with definitive diagnosis are considered. Although the hospital population of FBs in this study roughly reflects the general FB population (mostly in good health), this percentage is probably overestimated as a large majority of dog are presented to our institution for health issues. However, the objective of the current study was not to estimate the prevalence rate of neurological disorders in the general FB population, but rather among FBs presented to a veterinary hospital.

This study showed that the FB breed is affected by various neurological conditions, even if disorders of the central nervous system (CNS) were clearly predominant in this population. Hansen type I IVDH was by far the most prevalent neurological disease of FBs in this study, as it represented nearly half the overall conditions of the whole nervous system (45.5%), and 5.5% of all FBs presented to our institution during the study period. In a large American study evaluating inherited disorders in pure-breed and mixed-breed dogs, FB was found to be the second breed most frequently affected by IVDH after the Dachshund [8].

An important finding is that nearly 40% of IVDH in FBs from this study occurred in the cervical area. In other breeds, several studies demonstrated a lower percentage of cervical IVDH in comparison to thoracic-lumbar IVDH, rather located around 20–25%, especially in Dachshund [22–24]. However, an almost similar cervical versus thoracic-lumbar IVDH repartition has been reported in Beagle or Cocker Spaniel in a older study, even if the number of dogs in these breeds was limited [24].

Regarding IVDH position, the C3-C4 intervertebral space was the most frequently involved site, all locations combined. This contrasts either with recent [25] or older [26] findings indicating that C2-C3 was the most commonly affected disk in chondrodystrophic dogs presenting cervical IVDH, although only a small number of FBs were mentioned in the most recent report (*n* = 5). In the thoracolumbar area, T13-L1 was the most frequently affected site, followed by L2-L3 and L1-L2 in FB of our study. This is in partial agreement with previous publications revealing that the most often affected site in canine thoracolumbar IVDH was T12-T13, followed by T13-L1 and T11-T12 intervertebral spaces [27–29]. Note that the majority of dogs which constituted the populations in these previous studies were Miniature Dachshunds and not FBs. Conversely, IVDH were distributed more equitably between T13 and L4 in the FB reported here, which is consistent with a previous publication demonstrating that FB present thoracolumbar IVDH more caudally than Dachshund [28].

The median age of the FBs affected by cervical and thoracic-lumbar IVDH in the present study was 4.2 and 4.0 years respectively, which tends to be younger than in other breeds for both locations. Regarding Hansen type I thoracic-lumbar IVDH, this is indeed in contrast with the largest case series in which the estimated mean and median age is around 6 years old, all breeds combined [27–30]. Nonetheless, a single study comparing thoracolumbar Hansen type I IVDH in FB and Dachshunds showed that FBs were younger at the time of surgery [28], even if the number of FBs was limited in this report (*n* = 47). Similarly, studies addressing cervical IVDH revealed a median age ranging between 6 and 8 years old in chondrodystrophic dogs [25, 26, 31]. One hypothesis for this difference could be the fact that intervertebral disk degeneration occurs faster in FB than in other chondrodystrophic breeds. However, no study has yet compared the disk degeneration kinetic between chondrodystrophic breeds. Another recent study demonstrated that congenital vertebral malformations, common in FB, could promote intervertebral disk degeneration in the adjacent intervertebral spaces in chondrodystrophic breeds [32].

SAD was the second most frequent myelopathy and was identified in 25 dogs from this study, constituting the largest published population of FBs diagnosed with this condition. SAD was preponderant in the middle to caudal thoracic area in the FBs of this study. These findings regarding SAD location are consistent with a previous publication [7], the thoracic region being the most frequently affected in small chondrodystrophic breeds, such as Pug or FB. In addition, a potential underlying cause such as vertebral malformation or mild IVDH was identified in 64.0% of the cases of SAD in this study. This observation is also in agreement with the publication by Mauler and colleagues [7], which revealed that 61.5% of the 13 FBs reported in this study presented a concurrent spinal disorder. This could therefore predispose FB for acquired SAD in the thoracic area of the spinal cord. Compressive thoracic vertebral malformations resulting in a myelopathy were found in nearly 9% of FBs in this report, mainly represented by mid to caudal thoracic hemivertebrae. The true prevalence of vertebral malformation is however higher in FB, as asymptomatic dogs may present this abnormality [33], while only symptomatic FBs were included in the present study. A recent study showed indeed that vertebral malformations in neurologically normal FBs were detected in 93.5% of cases, and were more frequently observed than in other chondrodystrophic brachycephalic dog [34].

Encephalopathies were the second most frequently observed condition of the nervous system in this FBs population. Brain neoplasia appeared to be the primary cause of encephalopathy within this subpopulation, with glial tumour (either suspected or histologically confirmed) being the most frequent. This finding is in agreement with a past study, which revealed that FB, among other brachycephalic dogs, seems predisposed to gliomas [9]. Nevertheless, the diagnosis of glioma was not definitive in all cases in our study, as necropsy or biopsies were not performed in all animals presented with a brain tumour. In these cases, the diagnosis was based mainly on MRI characteristics. Even if several studies provided interesting MRI features giving indications for the differentiation in the tumour nature or subtype, these parameters remain to be improved, as they lack sensitivity and specificity [35–37]. The second most frequent encephalopathy was meningoencephalitis of unknown origin, which was found to be more common in small young to middle-aged female dogs according to a meta-analysis from 2010 [13]. In the present study too, FBs less than five and a half years old seemed more likely to be affected by MUO than other causes of encephalopathies, although the sensitivity for the selected cut-off value was not optimal. Similarly, females in the present population of FBs appeared to be more likely affected by this condition than male dogs, which is consistent with previous data [13].

Congenital deafness was the more frequently detected unclassified neurological disease, as 90.6% of FBs in this subpopulation were affected by this condition. Congenital deafness has been described in many canine breeds, including FB [38], and has been associated with the presence of white colour in coat or blue eyes in various breeds [38, 39]. Deaf FBs from our study were either white or contained white in their coat in nearly 80% of cases. The prevalence of bilateral deafness is usually lower than unilateral deafness according to several concordant studies [39–42]. Surprisingly, bilateral deafness was diagnosed more frequently than unilateral deafness in the FBs of this study, as 72.4% of the dogs were bilaterally deaf. This observation needs to be validated by other referral centres as several individuals were presented for auditory function screening purpose.

Otitis media/interna associated with peripheral vestibular syndrome was the most common disease in dogs presented with PNS/muscles disorders. This is in agreement with a previous study suggesting that the primary cause of peripheral vestibular syndrome in dog was otitis media/interna [43]. However, there was no information regarding the predisposition of a specific breed. To the authors’ knowledge, no information is available in the veterinary literature regarding the potential predisposition of a particular canine breed for otitis interna/vestibular neuritis. This disease might eventually spread to the overlying brain and/or meninges in some cases, thus resulting in bacterial meningoencephalitis [44].

The study period was specifically chosen after 2002 in order to minimize the measurement bias regarding conditions that required an MRI scan for accurate diagnoses. This date corresponds in fact to the arrival of a MRI device in our institution. However, a measurement bias may still exist because MRI scans were performed on a low-field device prior to 2013, whereas a high-field device was used after 2013. This may have affected the detection of certain disorders, such as MUO, immune-mediated myelitis or idiopathic epilepsy, even if a study in people revealed only subtle differences in the detection of brain lesions between low-field and high-field MRI devices [45]. Indeed, mild brain anomalies may be missed with a low-field MRI device, and a dog wrongly classified has having idiopathic epilepsy for example.

Finally, the FBs population in this study may differ from that encountered in other referral centres, as the number of insured dogs is probably higher in UK and North America than in France. This may have had a direct influence on case selection since it could have decreased the number of detailed clinical cases with a definitive diagnosis, and thus the prevalence rates of certain diseases.

## Conclusion

This is the first study addressing neurological conditions as a whole in a large cohort of FBs, and this further confirms the general impression of many veterinarians regarding the overall distribution of these disorders. Hansen type I IVDH appeared to be by far the most frequent neurological disease in middle-aged FBs, representing 5.5% of all FBs presented during the study period. FB tends to be more frequently affected by cervical IVDH than other breeds, and at a younger age. The topographical distribution indicates a greater tendency for involvement of the C3-C4 intervertebral disk.

SAD was the second most commonly diagnosed myelopathy, with an associated spinal abnormality in nearly two thirds of the cases. Suspected brain tumours and MUO were the most frequent encephalopathies, the latter preferentially affecting young to middle-aged female patients. Otitis interna with peripheral vestibular signs and bilateral congenital deafness associated with white coat, were also frequently observed, apart from CNS conditions. The high prevalence of various neurological diseases identified in this study might be explained by the specific body conformation of FB. This hypothesis needs however to be verified through comparative studies with other breeds, further multicentric studies in European and North American referral centres, and largest representative populations. Findings of the present study could be of interest for FBs owners and breeders, practicing veterinarians and pets insurance companies.

## Abbreviations

BAER: Brainstem auditory evoked response
CNS: Central nervous system
CSF: Cerebrospinal fluid
CT: Computed tomography
FB: French Bulldog
FLAIR: Fluid attenuated inversion recovery
IVDH: Intervertebral disk herniation
MRI: Magnetic resonance imaging
MUO: Meningoencephalitis of unknown origin
PNS: Peripheral nervous system
SAD: Spinal arachnoid diverticulum
